# On linear combinations of units with bounded coefficients and double-base digit expansions

**DOI:** 10.1007/s00605-012-0443-4

**Published:** 2012-10-06

**Authors:** Daniel Krenn, Jörg Thuswaldner, Volker Ziegler

**Affiliations:** 1 Institute of Optimisation and Discrete Mathematics (Math B), Graz University of Technology, Steyrergasse 30/II, 8010 Graz, Austria; 2 Chair of Mathematics and Statistics, University of Leoben, Franz-Josef-Strasse 18, 8700 Leoben, Austria; 3 Institute of Analysis and Computational Number Theory (Math A), Graz University of Technology, Steyrergasse 30/II, 8010 Graz, Austria

**Keywords:** Unit sum number, Additive unit structure, Digit expansions, 11R16, 11R11, 11A63, 11R67

## Abstract

Let $\mathfrak o$ be the maximal order of a number field. Belcher showed in the 1970s that every algebraic integer in $\mathfrak o$ is the sum of pairwise distinct units, if the unit equation $u+v=2$ has a non-trivial solution $u,v\in \mathfrak o ^*$. We generalize this result and give applications to signed double-base digit expansions.

## Introduction

In the 1960s Jacobson [7] asked, whether the number fields $\mathbb Q (\sqrt{2})$ and $\mathbb Q (\sqrt{5})$ are the only quadratic number fields such that each algebraic integer is the sum of distinct units. Śliwa [11] solved this problem for quadratic number fields and showed that even no pure cubic number field has this property. These results were extended to cubic and quartic fields by Belcher [2, 3]. In particular, Belcher solved the case of imaginary cubic number fields completely by applying the following criterion, which now bears his name, cf. [3].

### **Belcher’s criterion**

 Let $F$ be a number field and $\mathfrak o$ the maximal order of $F$. Assume that the unit equation

$$\begin{aligned} u+v=2, \qquad u,v\in \mathfrak o ^* \end{aligned}$$

has a solution $(u,v)\ne (1,1)$. Then each algebraic integer in $\mathfrak o$ is the sum of distinct units. 

The problem of characterizing all number fields in which every algebraic integer is a sum of distinct units is still unsolved. Let us note that this problem is contained in Narkiewicz’ list of open problems in his famous book [9, see page 539, Problem 18].

Recently the interest in the representation of algebraic integers as sums of units arose due to the contribution of Jarden and Narkiewicz [8]. They showed that in a given number field there does not exist an integer $k$, such that every algebraic integer can be written as the sum of at most $k$ (not necessarily distinct) units. For an overview on this topic we recommend the survey paper due to Barroero, Frei, and Tichy [1]. Recently Thuswaldner and Ziegler [13] considered the following related problem. Let an order $\mathfrak o$ of a number field and a positive integer $k$ be given. Does each element $\alpha \in \mathfrak o$ admit a representation as a linear combination $\alpha = c_1 \varepsilon _1 + \cdots + c_\ell \varepsilon _\ell$ of units $\varepsilon _1,\ldots ,\varepsilon _\ell \in \mathfrak o ^*$ with coefficients $c_i\in \{1,\ldots , k\}$ ? This problem was attacked by using dynamical methods from the theory of digit expansions. In the present paper we address this problem again. In particular, we wish to generalize Belcher’s criterion in a way to make it applicable to this problem.

In order to get the most general form, we refine the definition of the unit sum height given in [13].

### **Definition 1.1**

 Let $F$ be some field of characteristic 0, $\varGamma$ be a finitely generated subgroup of $F^*$, and $R\subset F$ be some subring of $F$. Assume that $\alpha \in R$ can be written as a linear combination

1.1 $$\begin{aligned} \alpha =a_1\nu _1 +\cdots +a_\ell \nu _\ell , \end{aligned}$$

where $\nu _1,\ldots ,\nu _\ell \in \varGamma \cap R$ are pairwise distinct and $a_1\ge \cdots \ge a_\ell >0$ are integers. If [in case there exists more than one representation of the form (1.1)] $a_1$ in (1.1) is chosen as small as possible, we call $\omega _{R,\varGamma }(\alpha )=a_1$ the $R$-$\varGamma$-*unit sum height of* $\alpha$. In addition we define $\omega _{R,\varGamma }(0):= 0$ and $\omega _{R,\varGamma }(\alpha ):= \infty$ if $\alpha$ admits no representation as a finite linear-combination of elements contained in $\varGamma \cap R$. Moreover, we define

$$\begin{aligned} \omega _\varGamma (R)=\max \{{\omega _{R,\varGamma }(\alpha ):}\quad {\alpha \in R}\} \end{aligned}$$

if the maximum exists. If the maximum does not exist we write

$$\begin{aligned} \omega _\varGamma (R)= \left\{ \begin{array}{ll} \omega&\quad \text{ if}\; \omega _{R,\varGamma }(\alpha )< \infty \quad \text{ for} \text{ each}\; \alpha \in R,\\ \infty&\quad \text{ if} \text{ there} \text{ exists} \; \alpha \in \text{ R} \text{ such} \text{ that} \; \omega _{R,\varGamma }(\alpha )= \infty . \end{array}\right. \end{aligned}$$

Let us note that for a number field $F$ with the group of units $\varGamma$ of an order $\mathfrak o$ of $F$ we have $\omega _\varGamma (\mathfrak o ) = \omega (\mathfrak o )$, where $\omega (\mathfrak o )$ is the unit sum height defined in [13].

With those notations our main result is the following.

### **Theorem 1.2**

 Let $F\subset \mathbb C$ be a field and $\varGamma$ a finitely generated subgroup of $F^*$ with $-1\in \varGamma$. Let $R$ be a subring of $F$ that is generated as a $\mathbb Z$-module by a finite set $\varepsilon \subset \varGamma \cap R$. Assume that for given integers $n\ge I \ge 2$ the equation

1.2 $$\begin{aligned} u_1+\cdots +u_I=n, \qquad u_1,\ldots ,u_I \in \varGamma \cap R \end{aligned}$$

has a solution $(u_1,\ldots ,u_I)\ne (1,\ldots ,1)$. Then we have $\omega _\varGamma (R) \le n-1$. 

The following section, Sect. 2, is devoted to the proof of Theorem 1.2. In the third section we apply our main theorem, Theorem 1.2, to some special orders of Shanks’ simplest cubic fields. A special case of that theorem yields applications to double-base expansions. There we choose $F=\mathbb Q$, $R=\mathbb Z$ and $\varGamma =\langle -1,p,q\rangle$, where $p$ and $q$ are coprime integers. We discuss that in Sect. 4.

## Proof of Theorem 1.2

We start this section by giving a short plan of the proof.

### *Plan of Proof*

 Let $\alpha \in R$ be arbitrary. Our goal is to find a representation of $\alpha$ of the form (1.1) in which the coefficients $a_1,\ldots , a_\ell$ are all bounded by $n-1$. We first show that $\alpha$ can be represented as a linear combination of the form (1.1) with $\nu _1,\ldots ,\nu _\ell$ chosen in a particular way. The idea of the proof is rather simple and is based on induction over the total weight of this representation (this is the sum of all of its coefficients, see Definition 2.2). Start with a representation of $\alpha$ as above and choose a coefficient which is greater than or equal to $n$ (if such a coefficient does not exist, we are finished). Now apply (1.2). This leads to a new representation of $\alpha$ of the form (1.1) whose total weight does not increase (and actually remains the same after excluding some trivial cases). This process is now repeated until we either have a representation in which all coefficients are bounded by $n-1$, or the support of the representation contains big gaps. In the first case we are finished. In the second case we can split the representation into two parts which are separated by a large gap. The total weight of each part is less than the total weight of the original representation of $\alpha$. We thus use the induction hypothesis on both of them, so we get a new representation of each part with coefficients bounded by $n-1$. Now, since the gap between the supports of these two parts is large, they do not overlap after we apply (1.2) to them in the appropriate way and we can put them together to find a representation as desired also in this case.

$$\square $$

Now we start with the proof of Theorem 1.2. First we introduce some notations. For integers $a$ and $b$ we write

$$\begin{aligned}{}[\![ {a},{b}]\!] := {\left\{ {a,a+1,\ldots ,b}\right\} } \end{aligned}$$

for the integers in the interval from $a$ to $b$. For tuples $\mathbf{x}={\left({x_1,\ldots ,x_M}\right)}$ and $\varvec{\varepsilon }={\left({{\varepsilon }_1,\ldots ,{\varepsilon }_M}\right)}$ we set

$$\begin{aligned} \varvec{\varepsilon }^\mathbf{x}:= {\varepsilon }_1^{x_1} \cdots {\varepsilon }_M^{x_M}. \end{aligned}$$

Observe first that each element of *R* has at least one representation of the form (1.1). The coefficients of that representation are integers, but not necessarily smaller than $n$.

### **A**

 There exists a $K$-th root of unity $\zeta$, elements $\eta _1,\ldots ,\eta _L \in \mathcal{E }$, and multiplicatively independent elements ${\varepsilon }_1,\ldots ,{\varepsilon }_M \in \varGamma \cap R$, abbreviated as $\varvec{\varepsilon }={\left({{\varepsilon }_1,\ldots ,{\varepsilon }_M}\right)}$, such that

$$\begin{aligned} u_i = \zeta ^{k_i} \varvec{\varepsilon }^\mathbf{r ^{(i)}}, \qquad i\in [\![ {1},{I}]\!], \end{aligned}$$

for some $k_1,\ldots ,k_I\in [\![ {0},{K-1}]\!]$ and some $\mathbf r ^{(1)},\ldots ,\mathbf r ^{(I)} \in \mathbb Z ^M$, each $\alpha \in R$ can be written as

$$\begin{aligned} \alpha = \sum _{k\in [\![ {0},{K-1}]\!]} \sum _{\ell \in [\![ {1},{L}]\!]} \sum _{\mathbf{x}\in \mathbb Z ^M} a_{k,\ell ,\mathbf{x}} \zeta ^k \eta _\ell \varvec{\varepsilon }^\mathbf{x}\end{aligned}$$

with non-negative integers $a_{k,\ell ,\mathbf{x}}$, and such that no relation of the form

$$\begin{aligned} \zeta ^{k} \eta _i \varvec{\varepsilon }^\mathbf{x}= \eta _j \varvec{\varepsilon }^\mathbf y , \quad i \ne j \end{aligned}$$

with integer exponents and $k\in \mathbb Z$ holds. 

### *Proof of A*

 Let $u_1,\ldots , u_I$ be as in (1.2). Choose a $K$-th root of unity $\zeta \in \varGamma \cap R$ (note that the torsion group of $\varGamma$ is finite and cyclic) and multiplicatively independent ${\varepsilon }_1,\ldots ,{\varepsilon }_M \in \varGamma \cap R$ with $M \le I$, such that

$$\begin{aligned} u_i = \zeta ^{k_i} {\varepsilon }_1^{r_1^{(i)}} \ldots {\varepsilon }_M^{r_M^{(i)}} = \zeta ^{k_i} \varvec{\varepsilon }^\mathbf{r ^{(i)}} \qquad (i\in [\![ {1},{I}]\!]) \end{aligned}$$

holds for some $\mathbf r ^{(1)},\ldots ,\mathbf r ^{(I)} \in \mathbb Z ^M$. We set

2.1 $$\begin{aligned} r:= \max \{r_m^{(i)}:\quad {i\in [\![ {1},{I}]\!],m\in [\![ {1},{M}]\!]}\} \end{aligned}$$

and want to mention that we reference to that $r$ later in this section.

Let us consider a finite subset ${\left\{ {\eta _1,\ldots ,\eta _L}\right\} } \subset \mathcal{E }$ such that all $\alpha \in R$ can be written as a linear combination

$$\begin{aligned} \alpha = \sum _{k\in [\![ {0},{K-1}]\!]} \sum _{\ell \in [\![ {1},{L}]\!]} \sum _{\mathbf{x}\in \mathbb Z ^M} a_{k,\ell ,\mathbf{x}} \zeta ^k \eta _\ell \varvec{\varepsilon }^\mathbf{x}\end{aligned}$$

with $a_{k,\ell ,\mathbf{x}}\in \mathbb Z$ (which is possible since $\mathcal{E }$ finitely generates $R$ as $\mathbb Z$-module). We can (and do) choose that finite subset such that no relation of the form

$$\begin{aligned} \zeta ^k \eta _i \varvec{\varepsilon }^\mathbf{x}= \eta _j \varvec{\varepsilon }^\mathbf y , \quad i \ne j \end{aligned}$$

with integer exponents $x,y \in \mathbb Z ^M$ and $k\in \mathbb Z$ holds.

Note that $\zeta ^k \eta _\ell \varvec{\varepsilon }^\mathbf{x}\in \varGamma \cap R$. Furthermore, we can choose the coefficients $a_{k,\ell ,\mathbf{x}}$ to be non-negative, since, by assumption, we have $-1\in \varGamma$, which allows us to choose the “signs” in our representation. $\square$

From now on we suppose that $\zeta$, $\eta _1,\ldots ,\eta _L$, and $\varvec{\varepsilon }$ are fixed and given as in **A**. We use the following convention on representations.

### **Convention 2.1**

 Let $\alpha \in R$ and suppose we have a representation of $\alpha$ where the coefficients are denoted by $a_{k,\ell ,\mathbf{x}}$ (small Latin letter with some index), i.e., $\alpha$ is written as

$$\begin{aligned} \alpha = \sum _{k\in [\![ {0},{K-1}]\!]} \sum _{\ell \in [\![ {1},{L}]\!]} \sum _{\mathbf{x}\in \mathbb Z ^M} a_{k,\ell ,\mathbf{x}} \zeta ^k \eta _\ell \varvec{\varepsilon }^\mathbf{x}\end{aligned}$$

We denote by $A \subset \mathbb Z ^M$ (capital Latin letter corresponding to the letter used for the coefficients) the minimal $M$-dimensional interval including all $\mathbf{x}$ with $a_{k,\ell ,\mathbf{x}} \ne 0$. We write

$$\begin{aligned} A = [\![ {\underline{A}_1},{\overline{A}_1}]\!] \times \ldots \times [\![ {\underline{A}_M},{\overline{A}_M}]\!]. \end{aligned}$$

We omit the range of the indices $k$ and $\ell$ since they are always the same. Thus $\alpha$ will be written as

$$\begin{aligned} \alpha = \sum _{k,\ell }\sum _{\mathbf{x}\in A} a_{k,\ell ,\mathbf{x}} \zeta ^k \eta _\ell \varvec{\varepsilon }^\mathbf{x}. \end{aligned}$$

An important quantity is the weight of a representation. It is defined as follows.

### **Definition 2.2**

 Let $\alpha \in R$ and suppose we have a representation as in **A**, i.e.,

$$\begin{aligned} \alpha = \sum _{k,\ell }\sum _{\mathbf{x}\in A} a_{k,\ell ,\mathbf{x}} \zeta ^k \eta _\ell \varvec{\varepsilon }^\mathbf{x}. \end{aligned}$$

with non-negative integers $a_{k,\ell ,\mathbf{x}}$. We call the minimum of all

$$\begin{aligned} \sum _{k,\ell } \sum _{\mathbf{x}\in A} a_{k,\ell ,\mathbf{x}} \end{aligned}$$

among all possible representations (as above) of $\alpha$ the *total weight of* $\alpha$ and write $w_\alpha$ for it. 

As mentioned in the plan of the proof of Theorem 1.2, we apply Eq. (1.2) to an existing representation to get another one. In the following paragraph, we define that replacement step, which will then always be denoted by $*$.

$*$ (Replacement step). Suppose we have a representation

$$\begin{aligned} \alpha = \sum _{k,\ell }\sum _{\mathbf{x}\in A} a_{k,\ell ,\mathbf{x}} \zeta ^k \eta _\ell \varvec{\varepsilon }^\mathbf{x}, \end{aligned}$$

where at least one coefficient $a_{k,\ell ,\mathbf{x}} \ge n$. We get a new representation by applying

$$\begin{aligned} u_1 + \cdots + u_I = n. \end{aligned}$$

More precisely, if $u_i = \zeta ^{k_i} \varvec{\varepsilon }^\mathbf{r ^{(i)}}$, then the coefficient $a_{k+k_i,\ell ,\mathbf{x}+\mathbf r ^{(i)}}$ is increased by $1$ for each $i \in [\![ {1},{I}]\!]$ and $a_{k,\ell ,\mathbf{x}}$ is replaced by $a_{k,\ell ,\mathbf{x}}-n$.

The following statements **B** and **C** deal with two special cases.

### **B**

 If $\alpha \in R$ with $w_\alpha < I$, then Theorem 1.2 holds. 

We use that statement as the basis of our induction on the total weight $w$.

### *Proof of B*

 Since $I \le n$ we have $w_\alpha < n$. So the sum of all (non-negative) coefficients is smaller than $n$. Therefore all coefficients themselves are in $[\![ {0},{n-1}]\!]$, which proves the theorem in that special case.$\square$

From now on suppose we have an $\alpha \in R$ with a representation

$$\begin{aligned} \alpha = \sum _{k,\ell } \sum _{\mathbf{x}\in A} a_{k,\ell ,\mathbf{x}} \zeta ^k \eta _\ell \varvec{\varepsilon }^\mathbf{x}, \end{aligned}$$

which has minimal weight. That means, we have $w := w_\alpha$.

### **C**

 If $I<n$, then Theorem 1.2 holds. 

### *Proof of C*

 Assume that there is a coefficient $a_{k,\ell ,\mathbf{x}} \ge n$ in the representation of $\alpha$. We apply $*$ to obtain a new representation. But because $I<n$, the new one has smaller total weight, which is a contradiction to the fact that $w$ was chosen minimal. $\square$

Because of **B** and **C** we suppose from now that $w \ge I$ and $I = n$. As indicated above, we prove Theorem 1.2 by induction on the total weight $w$ of $\alpha$. More precisely we want to prove the following claim by induction.

### **Claim 2.3**

 Assume that $\alpha \in R$ has a representation

$$\begin{aligned} \alpha = \sum _{k,\ell } \sum _{\mathbf{x}\in A} a_{k,\ell ,\mathbf{x}} \zeta ^k \eta _\ell \varvec{\varepsilon }^\mathbf{x}\end{aligned}$$

with non-negative integers $a_{k,\ell ,\mathbf{x}}$ and with minimal total weight $w$. Then $\alpha$ has also a representation of the form

$$\begin{aligned} \alpha = \sum _{k,\ell } \sum _{\mathbf{x}\in G} g_{k,\ell ,\mathbf{x}} \zeta ^k \eta _\ell \varvec{\varepsilon }^\mathbf{x}. \end{aligned}$$

with integers $g_{k,\ell ,\mathbf{x}} \in [\![ {0},{n-1}]\!]$ and where

$$\begin{aligned} G = [\![ {\underline{A}_1-f(w)},{\overline{A}_1+f(w)}]\!] \times \cdots \times [\![ {\underline{A}_M-f(w)},{\overline{A}_M+f(w)}]\!] \end{aligned}$$

with $$f(1)=0$$ and

$$\begin{aligned} f(w) = T(w) r+f(w-1)\qquad (w\in \mathbb N ), \end{aligned}$$

where

$$\begin{aligned} T(w) = \left(w+2(w-1)f(w-1)\right)^{Mw} K^w L^w. \end{aligned}$$

In order to prove Theorem 1.2 it is sufficient to prove Claim 2.3. As already mentioned, we use induction on the total weight $w$ of $\alpha$. Note that the induction basis has been shown above in **B**.

Let us start by looking what happens if one applies $*$.

### **D**

 Repeatedly applying $*$ yields pairwise “essentially different” representations of $\alpha$.

More precisely, by repeatedly applying $*$, it is not possible to get two representations

$$\begin{aligned} \alpha = \sum _{k,\ell } \sum _{\mathbf{x}\in A} a_{k,\ell ,\mathbf{x}} \zeta ^k \eta _\ell \varvec{\varepsilon }^\mathbf{x}= \sum _{k,\ell } \sum _{\mathbf{x}\in A} a_{k,\ell ,\mathbf{x}} \zeta ^k \eta _\ell {\varvec{\varepsilon }^{\mathbf{x}+\mathbf L }} \end{aligned}$$

with some $$\mathbf L \in \mathbb Z ^M \setminus {\left\{ \mathbf{0 }\right\} }$$. 

### *Proof of D*

 Remember that we assumed $I=n$. First, let us note that we have

$$\begin{aligned} n \le \sum _{i\in [\![ {1},{n}]\!]} \left|{u_i}\right| \end{aligned}$$

because of Eq. (1.2). Using the Cauchy–Schwarz inequality yields

$$\begin{aligned} n^2\le \left(\sum _{i\in [\![ {1},{n}]\!]} 1\cdot \left|{u_i}\right|\right)^2 \le n\sum _{i\in [\![ {1},{n}]\!]} \left|{u_i}\right|^2. \end{aligned}$$

Hence,

$$\begin{aligned} n<\sum _{i\in [\![ {1},{n}]\!]} \left|{u_i}\right|^2, \end{aligned}$$

unless $\left|{u_1}\right|=\cdots = \left|{u_n}\right|=1$ and $\sum _i u_i=n$, i.e., $u_1=\cdots =u_n=1$. Since the trivial solution has been excluded, we see that every application of $*$ makes the quantity

2.2 $$\begin{aligned} \sum _{k,\ell } \sum _{\mathbf{x}\in A} a_{k,\ell ,\mathbf{x}} \left( \left|{{\varepsilon }_1}\right|^{x_1} \cdots \left|{{\varepsilon }_M}\right|^{x_M}\right)^2 \end{aligned}$$

larger, i.e., the quantity (2.2) coming from coefficients $a_{k,\ell ,\mathbf{x}}^{\prime }$ is larger than (2.2) from $a_{k,\ell ,\mathbf{x}}$, where the $a_{k,\ell ,\mathbf{x}}^{\prime }$ are the coefficients after an application of $*$ on a representation with coefficients $a_{k,\ell ,\mathbf{x}}$. Note that the ${\varepsilon }_1,\ldots ,{\varepsilon }_M$ are fixed, cf. statement **A**.

Hence, repeatedly applying $*$ produces pairwise disjoint representations. Moreover, we cannot get the same representation up to linear translation in the exponents twice, i.e., we cannot get representations

$$\begin{aligned} \alpha = \sum _{k,\ell } \sum _{\mathbf{x}\in A} a_{k,\ell ,\mathbf{x}} \zeta ^k \eta _\ell \varvec{\varepsilon }^\mathbf{x}= \sum _{k,\ell } \sum _{\mathbf{x}\in A} a_{k,\ell ,\mathbf{x}} \zeta ^k \eta _\ell \varvec{\varepsilon }^{\mathbf{x}+\mathbf L } \end{aligned}$$

with $\mathbf L \in \mathbb Z ^M \setminus {\left\{ \mathbf{0 }\right\} }$. Such a relation would imply that $\varvec{\varepsilon }^\mathbf L =1$, which is a contradiction to the assumption that the ${\varepsilon }_1,\ldots ,{\varepsilon }_M$ are multiplicatively independent. $\square$

Now we look what happens after sufficiently many applications of $*$.

### **E**

 Set

$$\begin{aligned} T(w) := \left( w+2(w-1)f(w-1) \right)^{Mw} K^w L^w \end{aligned}$$

and suppose we have a representation

$$\begin{aligned} \alpha = \sum _{k,\ell } \sum _{\mathbf{x}\in A} a_{k,\ell ,\mathbf{x}} \zeta ^k \eta _\ell \varvec{\varepsilon }^\mathbf{x}. \end{aligned}$$

After at most $T(w)$ applications of $*$ we get a representation

$$\begin{aligned} \alpha = \sum _{k,\ell }\sum _{\mathbf{x}\in B} b_{k,\ell ,\mathbf{x}} \zeta ^k \eta _\ell \varvec{\varepsilon }^\mathbf{x}, \end{aligned}$$

such that one of the following assertions is true:

1. Each coefficient satisfies $b_{k,\ell ,\mathbf{x}} \in [\![ {0},{n-1}]\!]$ and
  $$\begin{aligned} \overline{B}_m - \underline{B}_m \le w + 2(w-1)f(w-1) \end{aligned}$$
  holds for all $m \in [\![ {1},{M}]\!]$.
2. There exists an index $m$ such that
  $$\begin{aligned} \overline{B}_m - \underline{B}_m > w + 2(w-1)f(w-1) \end{aligned}$$
  holds.

### *Proof of E*

 Each replacement step $*$ yields an essentially different representation, see **D**, and there are at most $T(w)$ possibilities to distribute our new coefficients in an interval $[\![ {0},{K-1}]\!]\times [\![ {1},{L}]\!]\times B$ with

$$\begin{aligned} \overline{B}_m - \underline{B}_m \le w + 2(w-1)f(w-1) \end{aligned}$$

for each $m$ with $1\le m\le M$. Therefore after at most $T(w)$ replacement steps we are either in case 1 or in case 2 of **E**. $\square$

### **F**

 With the setup and notations of **E**, a possible “translation of the indices” stays small.

More precisely, we have

$$\begin{aligned} \max \left\{ \left|{\underline{A}_m-\underline{B}_m}\right|:\quad {m\in [\![ {1},{M}]\!]}\right\} \le T(w) r, \end{aligned}$$

and

$$\begin{aligned} \max \left\{ \left|{\overline{A}_m-\overline{B}_m}\right|:\quad {m\in [\![ {1},{M}]\!]}\right\} \le T(w) r, \end{aligned}$$

where $r$ is as defined as in (2.1). 

### *Proof of F*

 The quantity $r$ is the maximum of all exponents in the representation of the $u_i$ as powers of the ${\varepsilon }_1,\ldots ,{\varepsilon }_M$. Thus, an application of $*$ can change the exponents, and therefore the upper and lower bounds, respectively, by at most $r$. We have at most $T(w)$ applications of $*$, so the statement follows. $\square$

Now we look at the two different cases of **E**. The first one leads to a result directly, whereas in the second one we have to use the induction hypothesis to get a representation as desired.

### **G**

 If we are in case (1) of **E**, then we are “finished”. 

### *Proof of G*

 Since

$$\begin{aligned} \left|\overline{A}_m - \overline{B}_m\right|\le T(w) r<T(w)r+f(w-1)=f(w) \end{aligned}$$

and

$$\begin{aligned} \left|\underline{A}_m - \underline{B}_m\right|\le T(w) r<T(w)r+f(w-1)=f(w) \end{aligned}$$

hold for each $m\in \mathbb N$ we have found a representation as desired in Claim 2.3. $\square$

### **H**

 If we are in case (2) of **E**, then we can split the representation into two parts and between them there is a “large gap”.

More precisely, there is a constant $c$ such that we can write $\alpha = \gamma + \delta$ with

$$\begin{aligned} \gamma = \sum _{k,\ell } {\mathop {\mathop {\sum }\limits _{\mathbf{x}\in B}}\limits _{x_m <c}} b_{k,\ell ,\mathbf{x}} \zeta ^k\eta _\ell \varvec{\varepsilon }^\mathbf{x}\end{aligned}$$

and

$$\begin{aligned} \delta = \sum _{k,\ell } {\mathop {\sum \limits _{\mathbf{x}\in B}}\limits _ {x_m >c + 2f(w-1)}} b_{k,\ell ,\mathbf{x}} \zeta ^k\eta _\ell \varvec{\varepsilon }^\mathbf{x}. \end{aligned}$$

### *Proof of H*

 In case 2 of **E** we have an index $m \in [\![ {1},{M}]\!]$ with

$$\begin{aligned} \overline{B}_m - \underline{B}_m \ge w + 2(w-1)f(w-1) \end{aligned}$$

The total weight of $\alpha$ is $w$, so the representation

$$\begin{aligned} \alpha = \sum _{k,\ell }\sum _{\mathbf{x}\in B} B_{k,\ell ,\mathbf{x}} \zeta ^k \eta _\ell \varvec{\varepsilon }^\mathbf{x}, \end{aligned}$$

has at most $w$ non-zero coefficients. Therefore, by the pigeon hole principle we can find an interval $J$ of length at least $2f(w-1)$ and with the property that all coefficients $a_{\mathbf{x}, i}$ fulfilling $x_m \in J$ are zero. Therefore we can split up $\alpha$ as mentioned. $\square$

### **I**

 If we have the splitting described in **H**, then Claim 2.3 follows for weight $w$. 

### *Proof of I*

 After renaming the intervals and coefficients, we have $\alpha = \gamma + \delta$ with

$$\begin{aligned} \gamma = \sum _{k,\ell } \sum _{\mathbf{x}\in C} c_{k,\ell ,\mathbf{x}} \zeta ^k\eta _\ell \varvec{\varepsilon }^\mathbf{x}\end{aligned}$$

and

$$\begin{aligned} \delta = \sum _{k,\ell } \sum _{\mathbf{x}\in D} d_{k,\ell ,\mathbf{x}} \zeta ^k\eta _\ell \varvec{\varepsilon }^\mathbf{x}. \end{aligned}$$

Both total weights $w_\gamma$ and $w_\delta$, respectively, are smaller than $w = w_\alpha$, so we can use induction hypothesis: We get representations

2.3 $$\begin{aligned} \gamma = \sum _{k,\ell } \sum _{\mathbf{x}\in E} e_{k,\ell ,\mathbf{x}} \zeta ^k\eta _\ell \varvec{\varepsilon }^\mathbf{x}\end{aligned}$$

with $$e_{k,\ell ,\mathbf{x}} \in [\![ {0},{n-1}]\!]$$ and

2.4 $$\begin{aligned} \delta = \sum _{k,\ell } \sum _{\mathbf{x}\in F} f_{k,\ell ,\mathbf{x}} \zeta ^k\eta _\ell \varvec{\varepsilon }^\mathbf{x}\end{aligned}$$

with $f_{k,\ell ,\mathbf{x}} \in [\![ {0},{n-1}]\!]$. The upper and lower bounds of the intervals in $C$ to $E$ differ by at most $f(w_\gamma ) \le f(w-1)$ in each coordinate. The same is valid for the intervals of $D$ to $F$. Since the intervals in $C$ and $D$ were separated by intervals of length at least $2f(w-1)$, therefore the intervals in $E$ and $F$ are disjoint. In other words, the two representations in (2.3) and (2.4) do not overlap. So we can add these two representations and obtain

$$\begin{aligned} \alpha = \sum _{k,\ell } \sum _{\mathbf{x}\in G} g_{k,\ell ,\mathbf{x}} \zeta ^k\eta _\ell \varvec{\varepsilon }^\mathbf{x}\end{aligned}$$

with $g_{k,\ell ,\mathbf{x}} \in [\![ {0},{n-1}]\!]$. We have

$$\begin{aligned} \max \left\{ \left|{\overline{G}_m-\overline{A}_m }\right|:\quad {m\in [\![ {1},{M}]\!]}\right\} \quad \le T(w) r+f(w-1) = f(w) \end{aligned}$$

and

$$\begin{aligned} \max \left\{ \left|{\underline{G}_m-\underline{A}_m}\right|:\quad {m\in [\![ {1},{M}]\!]}\right\} \le T(w) r+f(w-1) = f(w), \end{aligned}$$

which finishes the proof. $\square$

## The case of simplest cubic fields

Let $a$ be an integer and let $\alpha$ be a root of the polynomial

$$\begin{aligned} X^3-(a-1)X^2-(a+2)X-1. \end{aligned}$$

Then the family of real cubic fields $\mathbb Q (\alpha )$ is called the family of Shanks’ simplest cubic fields. These fields and the orders $\mathbb Z [\alpha ]$ have been investigated by several authors. In particular, in a recent paper of the second and third author [13] it was shown that the unit sum height of the orders $\mathbb Z [\alpha ]$ is $1$ in case of $a=0,1,2,3,4,6,13,55$ and the unit sum height $\le 2$ in case of $a=5$. Moreover, it was conjectured that $\omega (\mathbb Z [\alpha ])=1$ for all $a\in \mathbb Z$.

Using our main theorem we are able to prove the following result.

### **Theorem 3.1**

 We have $\omega (\mathbb Z [\alpha ])\le 2$ for all $a\in \mathbb Z$. 

### *Proof*

 First let us note some important facts on $\mathbb Q (\alpha )$ and $\mathbb Z [\alpha ]$, see for example Shanks’ original paper [10]. We know that $\mathbb Q (\alpha )$ is Galois over $\mathbb Q$ with Galois group $G=\{\text{ i}d, \sigma ,\sigma ^2\}$ and with $\alpha _2:=\sigma (\alpha )=-1-\frac{1}{\alpha }$. If we set $\alpha _1:= \alpha$, then $\alpha _1$ and $\alpha _2$ are a fundamental system of units. Now we know enough about the structure of $\mathbb Z [\alpha ]$ to apply Theorem 1.2.

If we can find three units $u_1,u_2,u_3\in \mathbb Z [\alpha ]^*$ such that $u_1+u_2+u_3=3$ and $u_i \ne 1$, then the theorem is a direct consequence of Theorem 1.2. Indeed we have

$$\begin{aligned} 3&= \overbrace{(\alpha _1^2+(-a+2)\alpha _1-a)}^{=u_1} \\&+ \overbrace{(-2\alpha _1^2+(2a-1)\alpha _1 +a+4)}^{=u_2} \\&+ \overbrace{(\alpha _1^2+(-a-1)\alpha _1-1)}^{=u_3} \\&= \alpha _1\alpha _2^2+\alpha _1^{-2}\alpha _2^{-1}+\alpha _1\alpha _2^{-1}. \end{aligned}$$

$$\square $$

## Application to signed double-base expansions

We start with the definition of a signed double-base expansion of an integer.

### **Definition 4.1**

 (*Signed double-base expansion*) Let $p$ and $q$ be different integers. Let $n$ be an integer with

$$\begin{aligned} n = \sum _{i\in \mathbb N _0, j\in \mathbb N _0} d_{ij} p^i q^j, \end{aligned}$$

where $d_{ij} \in {\left\{ {-1,0,1}\right\} }$ and only finitely many $d_{ij}$ are non-zero. Then such a sum is called a *signed* $p$-$q$-*double*-*base expansion of* $n$. The pair $(p,q)$ is called *base pair*. 

A natural first question is, whether each integer has a signed double-base expansion for a fixed base pair.

If one of the bases $p$ and $q$ is either $2$ or $3$, then existence follows since every integer has a *binary representation* (base $2$ with digit set ${\left\{ {0,1}\right\} }$) and a *balanced ternary representation* (base $3$ with digit set ${\left\{ {-1,0,1}\right\} }$), respectively. To get the existence results for general base pairs, we use the following theorem, cf. [5]

### **Theorem 4.2**

 (Birch) Let $p$ and $q$ be coprime integers. Then there is a positive integer $N(p,q)$ such that every integer larger than $N(p,q)$ may be expressed as a sum of distinct numbers of the form $p^iq^j$ all with non-negative integers $i$ and $j$. 

### **Corollary 4.3**

 Let $p$ and $q$ be coprime integers. Then each integer has a signed $p$-$q$-double-base expansion. 

Next we want to give an efficient algorithm that allows to calculate a signed double base expansion of a given integer. Birch’s theorem, or more precisely the proof in [5], does not provide an efficient way to do that. However, using our main result, there is a way to compute such expansions efficiently at least for certain base pairs.

### **Corollary 4.4**

 Let $p$ and $q$ be coprime integers with absolute value at least $3$. If there are non-negative integers $x$ and $y$ such that

4.1 $$\begin{aligned} 2 = \left|{p^x - q^y}\right|\!, \end{aligned}$$

then each integer has a signed $p$-$q$-double-base expansion which can be computed efficiently (there exists a polynomial time algorithm). In particular given a $p$-adic expansion of an integer $\alpha$, one has to apply (4.1) at most $O(\log (\alpha )^2)$ times. 

### *Proof*

 We start to prove the first part of the corollary and therefore apply Theorem 1.2 with $\mathbb F =\mathbb Q$, $R=\mathbb Z$ and $\varGamma$ is the multiplicative group generated by $-1,p$ and $q$. Since by assumption $2=\pm (p^x-q^y)$ we have a solution to (1.2) and Theorem 1.2 yields that $p$-$q$-double-base expansions exist.

Now let us prove the statement on the existence of a polynomial time algorithm. Assume that for the integer $\alpha$ the $p$-adic expansion

$$\begin{aligned} \alpha =a_{0}+a_{1}p+\cdots +a_{k} p^{k} \end{aligned}$$

is given, with $a_{0},\ldots ,a_{k}\in [\![ {0},{p-1}]\!]$. Let us note that the weight $w$ of this representation is at most $O(\log \alpha )$. Now the following claim yields the corollary. $\square$

### **Claim 4.5**

 Assume

$$\begin{aligned} \alpha =\sum _{i\in [\![ {0},{I}]\!]} a_i p^i \end{aligned}$$

with $a_i\in \mathbb Z$ and $I\in \mathbb N _0$, and set $w = \sum _{i\in [\![ {0},{I}]\!]} \left|{a_i}\right|$. Then, after at most $\frac{w^2-w}{2}$ replacement steps $*$ we arrive at a representation of the form

$$\begin{aligned} \alpha =\sum _{j\in [\![ {0},{J}]\!]} q^{jy} \sum _{k\in [\![ {0},{K}]\!]} b_{k,j}p^k, \end{aligned}$$

where the $b_{k,j}$ are integers with $\left|{b_{k,j}}\right| \le 1$, and $J,K\in \mathbb N _0$. 

### *Proof*

 We prove the claim by induction on $w$. If $w\le 1$ the statement of the claim is obvious. Further, if all the $a_i$ are in ${\left\{ {-1,0,1}\right\} }$ we are done. Therefore we assume that there is at least one index $i$ with $\left|{a_i}\right|>1$.

We now apply the replacement step $*$ in the following way: if $a_i>1$, then $a_i$ is replaced by $a_i-2$, if $a_i<-1$, then $a_i$ is replaced by $a_i+2$. After at most $w-1$ such steps, we get a new representation of the form

$$\begin{aligned} \alpha = \sum _{i\in [\![ {0},{I_c}]\!]} c_ip^i + q^y \sum _{i\in [\![ {0},{I_d}]\!]} d_ip^i, \end{aligned}$$

$I_c,I_d\in \mathbb N _0$, $c_i,d_i\in \mathbb Z$, such that all $c_i$ fulfil $\left|{c_i}\right|\le 1$. Note that no replacement step $*$ increases the weight $w$.

Now consider

$$\begin{aligned} \beta = \sum _{i\in [\![ {0},{I_d}]\!]} d_ip^i. \end{aligned}$$

The weight of $\beta$ fulfils

$$\begin{aligned} w_\beta = \sum _{i\in [\![ {0},{I_d}]\!]} \left|{d_i}\right| \le w-1, \end{aligned}$$

since in each replacement step it is increased exactly by $1$. Now, by induction, hypothesis we obtain a representation

$$\begin{aligned} \beta = \sum _{j\in [\![ {0},{J_e}]\!]} q^{jy} \sum _{k\in [\![ {0},{K_e}]\!]} e_{k,j}p^k, \end{aligned}$$

where the $e_{k,j}$ are integers with $\left|{e_{k,j}}\right| \le 1$ and $J_e,K_e\in \mathbb N _0$. Further, this can be done in $\frac{w_\beta ^2-w_\beta }{2}$ steps. Setting $b_{i,0} = c_i$ and $b_{i,k} = e_{i,k-1}$ for $k>0$ yields the desired representation. Moreover, this can be done with at most

$$\begin{aligned} \frac{w_\beta ^2-w_\beta }{2} + w-1 \le \frac{(w-1)(w-2)}{2} + w-1 = \frac{w(w-1)}{2} \end{aligned}$$

applications of $*$, which finishes the proof of the claim. $\square$

Now we want to give some examples for base pairs, where the corollary can be used.

### *Example 4.6*

 Let $(p,q)$ be a *twin prime pair*, i.e., we have $q=p+2$ and both $p$ and $q$ are primes. Then clearly

$$\begin{aligned} 2 = q - p, \end{aligned}$$

so, by Corollary 4.4, every integer has a signed $p$-$q$-double-base expansion, which can be calculated efficiently. 

### *Example 4.7*

 Let $p=5$ and $q=23$. We have

$$\begin{aligned} 2 = 5^2 - 23, \end{aligned}$$

therefore every integer has a signed $5$-$23$-double-base expansion, which can be calculated efficiently. Again Corollary 4.4 was used.

To see some concrete expansions, we calculated the following:

$$\begin{aligned} 995&= - 5^{5} + 5^{4} + 5^{3} \cdot 23 - 5^{2} + 5 \cdot 23 + 23^{2} + 1 \\ 996&= - 5^{3} + 5^{2} \cdot 23 + 23^{2} - 5 + 23 - 1 \\ 997&= - 5^{3} + 5^{2} \cdot 23 + 23^{2} - 5 + 23 \\ 998&= 5^{4} - 5^{3} - 5^{2} + 23^{2} - 5 - 1 \\ 999&= 5^{4} - 5^{3} - 5^{2} + 23^{2} - 5 \\ 1000&= 5^{4} - 5^{3} - 5^{2} + 23^{2} - 5 + 1 \\ 1001&= - 5^{3} + 5^{2} \cdot 23 + 23^{2} + 23 - 1 \\ 1002&= - 5^{3} + 5^{2} \cdot 23 + 23^{2} + 23 \\ 1003&= 5^{4} - 5^{3} - 5^{2} + 23^{2} - 1 \end{aligned}$$

In each case we started with an initial expansion, which is obtained by a greedy algorithm: For a $v\in \mathbb Z$ find the closest $5^i \cdot 23^j$, change the coefficient for that base, and continue with $v - 5^i \cdot 23^j$. Then we calculated the expansion by applying the equation $2 = 5^2 - 23$ as in the proof of Theorem 1.2. The implementation^1^ was done in Sage [12].

One can find pairs $(p,q)$ where Corollary 4.4 does not work. The following remark discusses some of those pairs. 

### *Remark 4.8*

 Consider the equation

4.2 $$\begin{aligned} 2 = \left|{p^x - q^y}\right| \end{aligned}$$

with non-negative integers $x$, $y$. A first example, where the corollary fails, is $p=5$ and $q=11$. Indeed, looking at Eq. (4.2) modulo 5 yields a contradiction. Another example is $p=7$ and $q=13$, where looking at (4.2) modulo $7$, yields a contradiction. A third example is $p=7$ and $q=11$.

So in the cases given in the remark above, as well as in a lot of other cases, we cannot use the corollary to compute a signed double-base expansion efficiently. This leads to the following question. 

### *Question 4.9*

 Is there an efficient (polynomial time) algorithm for each base pair $(p,q)$ to compute a signed $p$-$q$-double-base expansion for all integers?

There is also another way to use Theorem 1.2. For some combinations of $p$ and $q$ we can get a weaker result. First, we define an extension of the signed double-base expansion: we allow negative exponents in the $p^i q^j$, too. 

### **Definition 4.10**

 (*Extended signed double-base expansion*) Let $p$ and $q$ be different integers (usually coprime). Let $z\in \mathbb Q$. If we have

$$\begin{aligned} z = \sum _{i\in \mathbb Z , j\in \mathbb Z } d_{ij} p^i q^j, \end{aligned}$$

where $d_{ij} \in {\left\{ {-1,0,1}\right\} }$ and only finitely many $d_{ij}$ are non-zero, then we call the sum an *extended signed* $p$-$q$ *-double-base expansion of* $z$. 

With that definition, we can prove the following corollary to Theorem 1.2.

### **Corollary 4.11**

 Let $p$ and $q$ be coprime integers. If there are integers $a$, $b$, $c$, and $d$ with $(a,b,c,d)\ne (0,0,0,0)$ and such that

4.3 $$\begin{aligned} 2 = p^a q^b \pm p^c q^d, \end{aligned}$$

then every element of $\mathbb Z [1/p,1/q]$ has an extended signed $p$-$q$-double-base expansion which can be computed efficiently (polynomial time algorithm). 

### *Remark 4.12*

 If we have a solution to the equation in Corollary 4.4, then Corollary 4.11 works, too. But more can be said about the existence and efficient computability of extended double-base expansions for the elements of $\mathbb Z [1/p,1/q]$. If each integer has an efficient computable signed $p$-$q$-double-base expansion, then each element of $\mathbb Z [1/p,1/q]$ has an extended signed $p$-$q$-double-base expansion which can be computed efficiently. This result is not difficult to prove. 

Now we prove the corollary.

### *Proof of Corollary 4.11*

 The proof of this corollary runs along the same lines as the proof of Corollary 4.4.

We apply Theorem 1.2 with $\mathbb F =\mathbb Q$, $R=\mathbb Z [1/p,1/q]$ and $\varGamma$ is the multiplicative group generated by $-1$, $p$ and $q$. Since, by assumption, $2=\pm (p^aq^b-p^cq^d)$ we have a solution to (1.2), Theorem 1.2 yields that $p$-$q$-double-base expansions exist.

Next, we claim that we may assume $p$ and $q$ are odd and $p,q>3$. Indeed assuming that $p\in {\left\{ {2,3}\right\} }$, then we can write $\alpha \in \mathbb Z [1/p,1/q]$ in the form

$$\begin{aligned} \alpha = \frac{\widetilde{\alpha }}{p^{x_p}q^{x_q}} \end{aligned}$$

with $\widetilde{\alpha }\in \mathbb Z$ and appropriate exponents $x_p$ and $x_q$. Moreover, $\widetilde{\alpha }$ has a representation of the form

$$\begin{aligned} \widetilde{\alpha }=\sum _{i\in [\![ {0},{k}]\!]}a_i p^i \end{aligned}$$

with $a_i\in {\left\{ {-1,0,1}\right\} }$. However the computation of such a represenation can be done efficiently and takes polynomial time in the height $h(\alpha )$, where

$$\begin{aligned} h(n/m)=\max \{\log \left|{n}\right|, \log \left|{m}\right|, 1\} \end{aligned}$$

provided $n,m\in \mathbb Z$ are coprime.

Since we may assume $p,q>3$, we want to show next that a solution to Eq. (4.3) necessarly takes the form

$$\begin{aligned} 2=\pm p^{-a}\pm p^{-a}q^b, \end{aligned}$$

with $a,b\ge 0$. We observe that a solution to (4.3) with $a,c>0$ or $b,c>0$ does not exist, since otherwise $p\vert 2$ or $q\vert 2$. Next we note that if $a\ne c$ ($b\ne d$ respectively) the $p$-adic valuation ($q$-adic valuation) on the right hand side of (4.3) would be the minimum of $a$ and $c$ ($b$ and $d$ respectively) and in view of the left hand side, this minimum must be $0$. Thus any solution to Eq. (4.3) must be of one of the following forms:

$$\begin{aligned} 2&= \pm p^aq^b \pm 1, \\ 2&= \pm p^{-a}q^{-b} \pm p^{-a}q^{-b}, \\ 2&= \pm p^{-a}\pm p^{-a}q^b, \ \end{aligned}$$

or

$$\begin{aligned} 2&= \pm p^a\pm q^b, \end{aligned}$$

where $a$ and $b$ are positive integers. Obviously the first two cases have no solution and the last case has been treated in Corollary 4.4.

Now let us write $\alpha \in \mathbb Z [1/p,1/q]$ in the form

$$\begin{aligned} \alpha =\frac{a_0+a_1p+\cdots +a_kp^k}{q^{x_q}p^{x_p}}. \end{aligned}$$

We are now in a similar situation as in the proof of Corollary 4.4. Let $w=\sum _{i=1}^k \left|{a_i}\right|$. Then by similar arguments as in Corollary 4.4 we find an extended signed $p$-$q$-double-base expansion of $\alpha$ with at most $\frac{w^2-w}{2}$ applications of $*$. Thus we have a polynomial in $h(\alpha )$ time algorithm. $\square$

We can use the corollary proved above to get the following examples.

### *Example 4.13*

 Let $p$ be a *Sophie Germain prime* and $q=2p+1$. We obtain

$$\begin{aligned} 2 = q p^{-1} - p^{-1}. \end{aligned}$$

Using Corollary 4.11 yields that every element of $\mathbb Z [1/p,1/q]$ has an efficient computable extended signed $p$-$q$-double-base expansion.

The case when $p$ is a prime and $q=2p-1$ is a prime works analogously.

The end of this section is dedicated to a short discussion. All the results on efficient computability in this section needed a special representation of 2. We have given some pairs $(p,q)$ where the methods given here do not work.

Further, one could ask, whether the representations we get have a special structure. Of particular interest would be an algorithm to get expansions with a small number of summands (small number of non-zero digits). For a given base pair $(p,q)$ this leads to the following question 

### *Question 4.14*

 How to compute a signed $p$-$q$-double-base expansion with minimal weight for a given integer? 

A greedy approach for solving this question can be found in Berthé and Imbert [4], some further results can be found in Dimitrov and Howe [6].

## Footnotes

1 The source code can be found on http://www.danielkrenn.at/belcher/. Further a full list of expansions of the natural numbers up to $10,000$ can be found there.
